# LRRK2 A419V Is Not Associated with Parkinson's Disease in Different Chinese Populations

**DOI:** 10.1371/journal.pone.0036123

**Published:** 2012-07-10

**Authors:** Yih Ru Wu, Louis C. Tan, Xiaoli Fu, Chiung Mei Chen, Wing Lok Au, Ling Chen, Yi Chun Chen, Kumar M. Prakash, Yifan Zheng, Guey-Jen Lee-Chen, Yi Zhao, Jin-Sheng Zeng, Eng King Tan, Zhong Pei

**Affiliations:** 1 Department of Neurology, Chang Gung Memorial Hospital, Chang-Gung University College of Medicine, Taipei, Linkou, Taiwan; 2 Singapore General Hospital, National Neuroscience Institute, Singapore, Singapore; 3 Department of Neurology, The First Affiliated Hospital, Sun Yat-Sen University, Guangzhou, China; 4 Department of Life Science, National Taiwan Normal University, Taipei, Taiwan; 5 Duke-National University of Singapore, Graduate Medical School, Singapore, Singapore; Centre Hospitalier Universitaire Vaudois (CHUV), Switzerland

## Abstract

It has been suggested that a common LRRK2 polymorphic variant (A419V (rs34594498 C >T)) may be a risk factor among Asians (especially in Taiwan). In this study, we examined this variant in a larger and independent Taiwan cohort. We found the frequency of the variant (A419V) to be very rare in our Taiwan PD and controls (?0.6%). Further studies were conducted in two other Chinese populations (Singapore and China), comprising of a total of 3004 subjects including 1517 PD patients and 1487 control subjects. However, our multi-center Chinese study revealed that the frequency of the variant was rare (?0.4%) and was not associated with risk of PD, suggesting that the variant is not a major risk factor for PD among Chinese, at least in our study population.

## Introduction

Several pathogenic genes and susceptibility loci of Parkinson’s disease (PD) have been identified in both familial and sporadic cases in the last decade [1]. Mutations in the leucine-rich repeat kinase 2 gene (*LRRK2/PARK8*) were reported as a cause of PD in families with an autosomal dominant pattern of inheritance by two groups in 2004 [2], [3]. There has been considerable interest in *LRRK2,* probably due to the discovery of a common mutation (G2019S) [4] in many countries and its high frequency (30–40%) among Ashkenazi Jews and north African Arabs as well as the discovery of common risk variants (G2385R and R1268P) among Asians [5], [6].

Recently, investigators in a multi-center study genotyped 121 exonic *LRRK2* variants in three ethnic series (white, Asian, and Arab–Berber), and identified a common polymorphic risk variant (A419V (rs34594498 C >T)) in 3 Asian cohorts (1376 Asian patients and 962 controls) [7]. Remarkably, the variant A419V conferred an increased risk in developing PD with an odds ratio of >7.0 in one cohort consisting of ethnic Chinese from Taiwan [7]. Currently, it is unclear whether this observation is specific to ethnic Chinese or this risk factor applies to Asians in general.

## Results

The mean age at onset of PD in the three groups (Taiwan, Singapore and China) was 63.0±11.2, 64±10, 60.1±11.0 years, and the mean age of control subjects at recruitment was 60.1±11.2, 59.9±9.8 and 60.2±13.2 years. The gender of the patient and control groups was matched in each cohort. All three cohorts showed similar minor allele frequency (MAF) of the SNP in the normal population (the frequency of 200 cases in Singapore was previously reported to be 0.4%) [8], but a marginal higher MAF was found in the PD group of the China cohort (Table 1).

**Table 1:** Frequency of Allele Polymorphisms of *LRRK2* rs34594498 among Parkinson’s Disease (PD) and Controls in Taiwan, Singapore and China.

	PD (%)	Controls (%)	OR (95% CI)	*P*-value
Subjects of Taiwan (T)	N = 485	N = 494		
Common (C) allele	966 (99.59 )	982 (99.39)	1.00	
Minor (T) allele	4 (0.41 )	6 (0.61 )	0.6777 (0.19–2.41)	0.57
Subjects of Singapore (S)	N = 560	N = 550		
Common (C) allele	1116 (99.64)	1096 (99.64)	1.00	
Minor (T) allele	4(0.36)	4 (0.36)	0.98 (0.24–3.93 )	0.98
Subjects of China (C)	N = 472	N = 443		
Common allele (C)	939 (99.48)	883 (99.67)	1.00	
Minor allele (T)	5 (0.52 )	3 (0.33)	1.56 (0.37–6.5 )	0.56
Combined subjects (T+S+C)	N = 1517	N = 1487		
Common allele (C)	3021 (99.58)	2961(99.56 )	1.00	
Minor allele (T)	13 (0.42)	13 (0.44 )	0.98 (0.45–2.18 )	0.96

All p-values were calculated by means of chi-squared test.

P-values and OR calculated in relation to major allele G.

## Discussion

Our multi-center Chinese study, with a much larger sample size (>3000) than the previously reported Taiwan Chinese cohort (about 700) or combined mixed Asian cohort (>2300), showed the frequency of the variant was rare (?0.4%) and was not associated with risk of PD, suggesting that the variant is not a major risk factor for PD among Chinese, at least in our study population.

It’s intriguing why the results of ours and the other Taiwanese group in the multi-center study were markedly different [7]. Possible reasons may be due to very low MAF (0.33–1.9%) of the exonic variant (A419V), natural sampling variation, and population heterogeneity. The potential biological functions for this SNP are unknown. A419V is near the N-terminal region of the *LRRK2* protein and maybe functionally relevant to disease development, or it is in linkage disequilibrium with a non coding variant or the actual causative variant. As new loci susceptible to different complex diseases are continuously being discovered in genome-wide association and whole-genome sequencing studies, the results of our study demonstrate the importance of revisiting loci at which common or rare variants have been identified. It is possible that the A419V variant is a risk factor in only selected Asian populations or even subset of Chinese, unlike G2385R which has been consistently seen across all Chinese populations [5]. Other factors such as gene-gene, gene-environmental factors may be confounding variables to explain the divergent associations observed. Further multi-center studies will be warranted to characterize the frequency of the variant in different populations, in order to better decipher its contribution to the disease pathogenesis.

## Materials and Methods

### Ethics Statement

All subjects gave written informed consent for this study under a protocol approved by local hospital internal ethics and scientific boards. (Chang Gung Memorial Hospital internal ethics and scientific boards for the Taiwanese cohort, Singapore General Hospital and Singhealth ethics and scientific boards for the Singapore cohort and The First Affiliated Hospital internal ethics and scientific boards, Sun Yat-Sen University for the China cohort).

### Subjects

The diagnosis of PD was based on the UK PD Society Brain Bank clinical diagnostic criteria (more than one affected relative was excluded). Unrelated healthy adult volunteers matched for age, gender, ethnic origin, and area of residences were recruited as controls. We first recruited 485 PD patients and 494 control subjects in the Taiwanese Cohort and we found the frequency of the variant (A419V) to be very rare (∼0.6%). The subjects examined here did not overlap with the Taiwanese population included in the study performed by Ross et al [7]. Therefore, additional replications were conducted in two other Chinese population (Singapore and China), comprising of a total of 3004 subjects including 1517 PD patients and 1487 control subjects subsequently to be analyzed.

### Genotyping

TaqMan® Assays were used for SNP genotyping analysis in the Taiwanese cohort. Genotyping was carried out with mass spectrometric analysis using the Sequanom^R^ and sequence analysis from Singapore, and direct sequence analysis in the China cohort.

### Statistical Analyses

Chi square test was used to compare the frequency of the allele in both cases and controls. The genotypes of the case and controls followed the Hardy Weinberg equilibrium. Odds ratios and 95% confidence intervals were estimated. A free and open source software (OpenEpi) was used for the statistical analysis [9].
